# A Sensitive IHC Method for Monitoring Autophagy-Specific Markers in Human Tumor Xenografts

**DOI:** 10.1155/2016/1274603

**Published:** 2016-05-10

**Authors:** Helen He, Yu Yang, Zhongmin Xiang, Lunyin Yu, Jouhara Chouitar, Jie Yu, Natalie Roy D'Amore, Ping Li, Zhi Li, Douglas Bowman, Matthew Theisen, James E. Brownell, Stephen Tirrell

**Affiliations:** ^1^Department of Molecular Pathology, Takeda Pharmaceuticals International Co., Cambridge, MA 02139, USA; ^2^Cancer Pharmacology, Takeda Pharmaceuticals International Co., Cambridge, MA 02139, USA; ^3^Early Discovery, Takeda Pharmaceuticals International Co., Cambridge, MA 02139, USA; ^4^Protein Sciences, Takeda Pharmaceuticals International Co., Cambridge, MA 02139, USA

## Abstract

*Objective.* Use of tyramide signal amplification (TSA) to detect autophagy biomarkers in formalin fixed and paraffin embedded (FFPE) xenograft tissue.* Materials and Methods.* Autophagy marker regulation was studied in xenograft tissues using Amp HQ IHC and standard IHC methods.* Results.* The data demonstrate the feasibility of using high sensitivity TSA IHC assays to measure low abundant autophagy markers in FFPE xenograft tissue.

## 1. Introduction

Autophagy is a catabolic process that targets aged or malfunctioning organelles and damaged or misfolded proteins for lysosomal degradation. There are several autophagy-related proteins whose coordinated function is required for conjugation of the autophagy-specific ubiquitin-like proteins (Ubls), LC3A, LC3B, and LC3C, and GABARAP, GABARAP-L1, and GATE-16 to the lipid phosphatidylethanolamine (PE). Together with adapter proteins, including p62 and NBR1, these lipidated Ubls help to encapsulate substrates into double-membrane vesicles called autophagosomes which ultimately fuse with lysosomes. At basal levels, this process plays an important role in developing and maintaining cellular homeostasis and genomic integrity [1]. Autophagy may also contribute to tumorigenesis by promoting cell survival in response to metabolic or oncogenic stress and to resistance to chemotherapy [2–4]. Recent studies have shown that the pharmacological and/or genetic inhibition of autophagy can sensitize cancer cells to the lethal effects of various cancer therapies, including chemotherapy, radiotherapy, and targeted therapies [5, 6]. These results suggest that inhibition of autophagy may provide a valuable sensitizing strategy for cancer treatments.

It is well documented that nutrient starvation results in mTOR inhibition and induction of autophagy [7]. As a central regulator of cell growth, mTOR plays a key role at the interface of the pathway that regulates the balance between cell growth and autophagy in response to nutritional states, growth factors, and stress signals. Hypoxia is also known to induce autophagy in highly proliferating tumor cells [8] and the adverse effects of hypoxia to chemotherapy treatment have been well documented [3, 9, 10]. The recent finding that hypoxia results in mTOR pathway inhibition and autophagy upregulation may contribute to this self-protective mechanism in certain cancers [11].

Lipid conjugation of the autophagy Ubls is essential for autophagosome formation and is mediated by the E1 activating enzyme ATG7 along with the E2 conjugating enzyme ATG3 and an E3 ligase complex consisting of ATG5–ATG12/ATG16 [12]. ATG7 knockdown reduces autophagy Ubl lipidation and slows down basal constitutive autophagy process resulting in accumulation of the autophagy adapter proteins p62 and NBR1. These proteins, which function to deliver polyubiquitinated, misfolded, or aggregated proteins and dysfunctional organelles to autophagosomes, are also themselves autophagy substrates [13–15]. Thus, monitoring the cellular levels and localization of the autophagy Ubls and adapter proteins can be used as a measure for autophagy activation or inhibition.

Methods to study autophagy regulation in cells* in vitro* have often employed detection of GFP-labeled autophagy proteins such as LC3B or electron microscopy to observe double membrane-bound autophagosomal structures. However, these approaches are not ideal for monitoring autophagy regulation in an* in vivo* drug discovery setting [16]. An alternative approach such as IHC would be valuable for assessing autophagy regulation in tissues in clinically relevant settings. Unfortunately, significant technical challenges exist, as conventional polymer based IHC methods are largely ineffective for detecting autophagy-specific markers due to the low abundance and transient nature of autophagosomes [16–20].

To address these issues, tyramide signal amplification (TSA) technology was applied to the autophagy markers LC3B and NBR1, which resulted in a more robust and measurable signal as compared to the conventional polymer IHC platform. TSA technology is based on the catalyzed reporter deposition (CARD) principle [21]. Using the peroxidase activity of an HRP-conjugated secondary antibody, labeled-tyramide molecules are deposited in the vicinity of the antigen. The label could be a chromophore, a hapten, or an enzyme such as HRP, and the signals are then detected accordingly. In the scheme used in the present study, HQ (a hapten) was attached to tyramide and later detected using an HRP-conjugated anti-HQ antibody.

Using this methodology, autophagy-related markers were monitored under conditions of autophagy induction and inhibition in Calu-6 and HCT116 xenograft tumor models. MLN0128 is a potent mTOR kinase inhibitor that induces autophagy by inhibiting the mTOR pathway [22]. In contrast, xenograft tumors in which ATG7 was knocked down using shRNA have reduced levels of Ubl lipidation and impaired autophagy. Using these methods to manipulate autophagy, robust changes were observed in both LC3B and NBR1 levels in a time-dependent manner using the sensitive TSA-mediated detection method. This enabled the measurement of dynamic changes in autophagy regulated proteins without the use of engineered artificial protein labeling. Using this method, the correlation of different markers in a multiplex format was examined and the relationship between basal levels of hypoxia and autophagy in tumor development was monitored. The methodology used herein should provide a valuable approach to studying autophagy regulation in various disease settings.

## 2. Materials and Methods

### 2.1. Generation of HCT116 Cell Lines with Doxycycline-Inducible ATG7 Knockdown

ATG7 shRNA, TRCN0000007587 (Sigma-Aldrich), was cloned into the doxycycline-inducible pTRIPZ lentiviral vector (Open Biosystem RHS4750). Lentivirus was packaged in 293T cells using the MISSION Lentiviral Packaging Mix (Sigma-Aldrich SHP001) and viral supernatants were collected at 48 and 72 hours after transfection and used to transduce HCT116 cells in the presence of 8 *μ*g/mL polybrene (Sigma-Aldrich 107689) for 72 hours. The cells were then selected for 8 days in the presence of 1 *μ*g/mL puromycin (Invitrogen A1113803). Single cell clone, ATG7 shRNA3.3, was isolated by limiting dilution cloning. Doxycycline (Sigma-Aldrich D9891) induced ATG7 knockdown in the ATG7 shRNA3.3 clone was confirmed by Western blot using anti-ATG7 antibody (Epitomics, 2054-1). Scramble control line F6 was generated using the nonsilencing control shRNA in pTRIPZ vector (Open Biosystem RHS4743) alongside with the same process.

### 2.2. Doxycycline-Inducible ATG7 Knockdown Xenograft Model

HCT116 cell lines with doxycycline-inducible ATG7-shRNA (Clone 3.3) were propagated in the cell culture medium, McCoy's 5A, with 10% fetal bovine serum (FBS). Cells were grown and expanded for the inoculation of 2 × 10^6^ cells in 0.1 mL per mouse. Using a 26 × 3/8 gauge needle, 0.1 mL cell suspension in media-free PBS was subcutaneously inoculated into the right dorsal flank area of the female NCr nude mice. When the tumors reached approximately 200 mm^3^, by caliper measurements (typically around 10 days after inoculation), the mice were randomized to receive food with or without doxycycline (0.0625 mg/kg) for 7, 14, or 21 days and then sacrificed.

### 2.3. mTOR Inhibitor, MLN0128, Treatment Animal Model

Calu-6 human lung cancer cells were propagated in cell culture medium MEM with the addition of 10% FBS. Cells were grown and expanded for inoculation of 5 × 10^6^ cells in 0.1 mL per mouse. Using a 26 × 3/8 gauge needle, 0.1 mL cell suspension in media-free PBS solution was subcutaneously inoculated into the right dorsal flank area of female nude mice. Approximately 3 weeks after inoculation, when the tumor size reached 500–800 mm^3^, animals were randomized into treatment groups. All animals received a 0.2 mL single oral dose of MLN0128 (5.0 mg/kg). At the specific time point (30 minutes, 1, 2, 4, 8, and 24 hours) after dose, the animals were sacrificed. Tumors were collected and fixed in 10% neutral buffered formalin (NBF) before processing for histology studies.

### 2.4. Western Blot

Tumor tissues were processed in a Covaris E200 tissue homogenizer in accordance with the manufacturer's instructions. RIPA lysis buffer (Millipore) with 1x protease inhibitor cocktail (Calbiochem), 2 mmol/L sodium orthovanadate (Sigma-Aldrich), 25 mmol/L sodium fluoride, and 25 mmol/L *β*-glycerophosphate was used. Cold lysis buffer (300 to 800 *μ*L) was added to each tumor sample followed by sonication. The supernatants were transferred to new tubes and protein concentrations determined by SpectraMax Plus 384 (Molecular Devices). For Western blot analysis, tumor lysates (20 *μ*g protein) were loaded onto 4% to 12% gradient Bis-Tris gels (Invitrogen) and subjected to electrophoresis. Separated proteins were transferred to PVDF-FL membranes (Millipore) using a semidry transfer apparatus. The membranes were blocked with LI-COR blocking buffer (LI-COR Biosciences, 927-40000) and then incubated with primary antibody in LI-COR blocking buffer plus 0.1% tween-20 (Sigma-Aldrich, P 1397) overnight at 4°C. After three washes with TBST, the membranes were incubated with Alexa Fluor® 680-labeled goat anti-rabbit IgG (Life Technology) in LI-COR blocking buffer plus 0.1% tween-20 for 1 hour. While protected from light, the membranes were washed five times with TBST, once with TBS, and then dried. The membranes were scanned with the Odyssey Infrared Imaging System (LI-COR Biosciences) and the band intensities were quantified with Odyssey software. The following primary antibodies were used: monoclonal rabbit anti-LC3B (Cell Signaling Technology, 3868, 412 *μ*g/mL, 1 : 1000); polyclonal rabbit anti-SQSTM1/P62 antibody (Abcam, 91526, 1 *μ*g/mL, 1 : 1000); and polyclonal rabbit anti-ATG7 (Epitomics, 2054-1, 1 : 1000).

### 2.5. Heavy Membrane Fraction

Tumor tissues were processed in a Covaris E200 tissue homogenizer in accordance with the manufacturer's instructions. Heavy membrane lysis buffer (250 mM mannitol, 70 mM sucrose, 0.5 mM EGTA, and 5 mM HEPES-NaOH, pH 7.2) with 1x protease inhibitor cocktail (Calbiochem), 2 mmol/L sodium orthovanadate (Sigma-Aldrich), 25 mmol/L sodium fluoride, and 25 mmol/L *β*-glycerophosphate (Sigma-Aldrich) was used. Cold heavy membrane lysis buffer (1000 *μ*L) was added to each tumor sample followed by sonication. Lysates were transferred to Eppendorf tubes and centrifuged at 3,000 rpm in Eppendorf Centrifuge 5417R at 4° for 10 minutes, and supernatants were transferred to new Eppendorf tubes. After taking 250 *μ*L of supernatant and saving as total lysate l (TL1), the remaining supernatants were centrifuged at 14,000 rpm at 4° for 15 minutes. The resulting pellets were labeled as heavy membrane fraction (HMF). The HMF pellets were lysed in 150 *μ*L of RIPA lysis buffer with the previously mentioned inhibitors and kept on ice for 20 minutes. Protein concentrations were determined for both TL1 and HMF fractions using the BCA method.

### 2.6. Standard Immunohistochemistry (IHC)

All xenograft tumor samples were fixed in 10% NBF for a minimum of 16 hours before being dehydrated and embedded in paraffin (FFPE). For IHC evaluations, 5 *μ*m sections of FFPE tissue were mounted onto glass slides, dried overnight at room temperature, and baked at 60°C for 1 hour prior to use. Tumor sections were processed by automated staining using either the Discovery XT (Ventana Systems) or the Bond RX (Leica Biosystems) autostainers, following preset protocols. In brief, the sections were subjected to ethylenediaminetetraacetic acid (EDTA) based antigen retrieval for 20 minutes and protein block (Dako, DS9390) for 12 minutes before incubation with primary antibody for 1 hour at room temperature. The following primary antibodies were used: polyclonal rabbit anti-carbonic anhydrase IX (CAIX) antibody (Novus Biologicals, NB100-417, 1 : 2500); polyclonal rabbit anti-SQSTM1/P62 antibody (Abcam, 91526, 1 : 5000); polyclonal rabbit anti-ATG7 (Epitomics, 2054-1, 1 : 800); monoclonal rabbit anti-pS6 (Cell Signaling Technology, 5364, 1 : 2500); monoclonal rabbit anti-LC3B (Cell Signaling Technology, 3868, 1 : 50); and monoclonal rabbit anti-NBR1 (inhouse antibody, 1 : 50; validation of ML85-61-3 data not shown). 3,3-Diaminobenzidine DAB chromogenic detection kits, OmniMap HRP Multimer kit (Ventana Systems, 760-159) or Bond Polymer Refine Detection kit (Leica Biosystems, 9800 DAB and DS9390 Red), were used following the manufacturer's instructions.

Colocalization studies of CAIX and pS6 used sequential standard IHC detection protocols on BOND RX (Leica Biosystems) with both Bond Polymer Refine Detection DAB and Red kits.

### 2.7. Amp HQ IHC

For detecting low abundance antigens LC3B and NBR1, DISCOVERY Amp HQ Kit (Ventana Systems, 760-052), a tyramide signal amplification detection kit, was used. FFPE sections were dried overnight at room temperature and then baked at 60°C for 1 hour prior to loading onto Discovery XT autostainer (Ventana Systems). The sections were subjected to EDTA-based CC1 mild antigen retrieval for 20 minutes and protein block (Dako, DS9390) for 12 minutes before incubation with primary antibody for 1 hour at room temperature. The LC3B primary antibody (Cell Signaling Technology, 3868, 412 *μ*g/mL, 1 : 400) and NBR1 (Takeda Pharmaceuticals Inc., rabbit monoclonal antibody, 1.7 mg/mL, 1 : 2000) were used for this study. Following primary antibody incubation, the Amp HQ kit in conjunction with (1) anti-HQ Multimer, (2) OmniMap HRP Multimer, and (3) Chromogenic Detection Kit was used following the manufacturer's instructions (Ventana Systems).

### 2.8. Image Analysis

The IHC-stained slides were scanned by Aperio ScanScopeXT (Leica Biosystems) using the 20x objective, and the digital images were stored in eSlide Manager (Leica Biosystems) database. Aperio Genie (Leica Biosystems), a pattern recognition tool, was used to select viable areas within the tumor. For CAIX-LC3B analysis, CAIX-positive and CAIX-negative regions were manually annotated and transferred to the LC3B image for subsequent image analysis. Whole slide image analysis was performed using positive pixel count algorithm where the area of positive signal was measured and normalized to the corresponding total viable tumor area. All values are expressed as the mean ± SD. One-way ANOVA was used for statistical analysis with a significance level set at *p* < 0.05.

## 3. Results

### 3.1. Detection of Autophagy Adaptor Molecules in Xenograft Tissue Using Amp HQ

**Figure 1:**
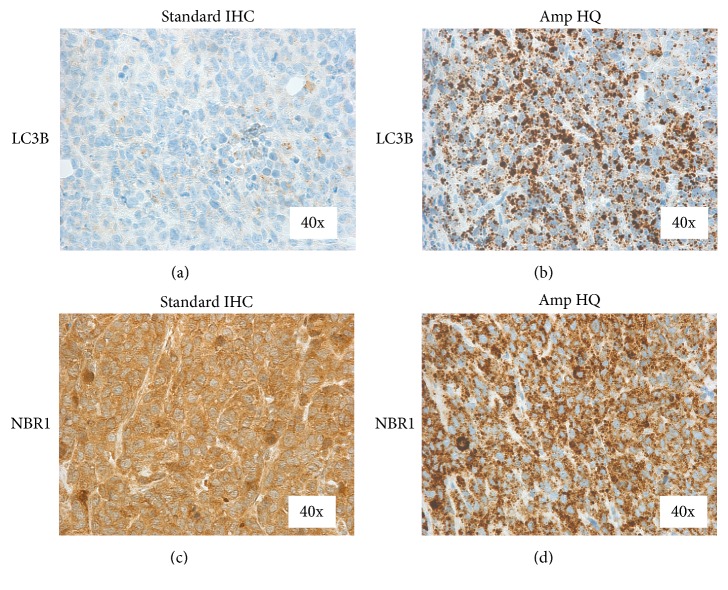
Comparison of standard IHC with Amp HQ for detection of LC3B and NBR1. Sections of Calu-6 xenograft tumor from nude mice treated with mTOR inhibitor MLN0128 were stained with rabbit monoclonal antibodies raised against LC3B and NBR1. (a) LC3B signal detected by standard OminiMap IHC showed very fine, lightly stained puncta in the tumor cell cytoplasm. (b) LC3B signal detected by Amp HQ IHC showed pronounced dark stained puncta in the cytoplasm. (c) NBR1 signal detected by standard OminiMap IHC showed very fine puncta in the tumor cell cytoplasm. (d) NBR1 signal detected by Amp HQ IHC showed dark stained puncta in the tumor cell cytoplasm.

The use of standard IHC methods for detecting and quantifying autophagy-specific biomarkers has been limited due to the low abundance and transient nature of autophagosomes. With standard IHC methods, signal is typically amplified with antispecies multimer complexes containing multiple enzyme molecules. FFPE Calu-6 xenograft tumors stained for LC3B and NBR1 with standard IHC methods showed diffuse brown staining in the cytoplasm with very fine, light staining puncta structures (Figures 1(a) and 1(c)). In contrast, Amp HQ uses a tyramide substrate-based amplification where in the presence of hydrogen peroxide, and immobilized HRP converts tyramide substrate into a reactive intermediate that covalently binds to electron rich regions of proteins immediately adjacent to the activating HRP enzyme. Subsequent detection of the HQ substrate yields large amplification of signal. Using the Amp HQ method on Calu-6 xenograft sections, both LC3B and NBR1 were clearly detected as dark-staining puncta structures in the cytoplasm of viable tumor (Figures 1(b) and 1(d)). To verify the Amp HQ assays were capable of measuring specific changes to LC3B and NBR1 levels under conditions of autophagy induction and inhibition, the autophagy pathway was manipulated* in vivo* using the investigational mTOR inhibitor, MLN0128, to activate autophagy and using doxycycline-inducible ATG7shRNA knockdown to downregulate autophagy.

### 3.2. Upregulation of Autophagy Pathway Observed with mTOR Pathway Inhibition

**Figure 2:**
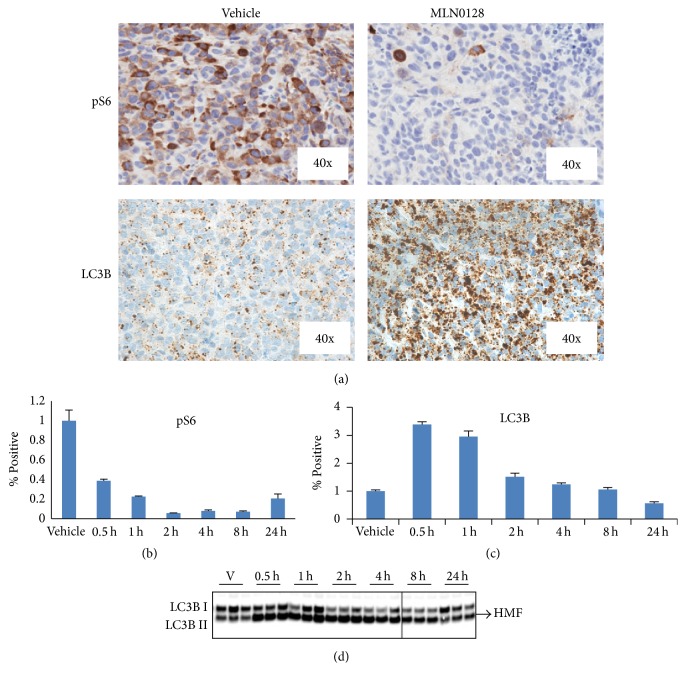
Sections of HCT116 xenograft tumor from nude mice treated with vehicle or investigational mTOR inhibitor MLN0128 were stained with rabbit monoclonal anti-pS6 and anti-LC3b antibodies. (a) Standard IHC staining of pS6 in vehicle-treated xenograft samples showed strong pS6 signal, whereas standard IHC staining of pS6 in MLN0128 treated xenograft (5 mg/kg for 1 hour) samples showed reduced pS6 levels compared to vehicle-treated samples. Amp HQ staining of LC3B in sections of vehicle-treated xenograft samples showed low levels of LC3B puncta staining predominately in the tumor cell cytoplasm, whereas Amp HQ staining of LC3B in sections of MLN0128-treated xenograft (5 mg/kg for 1 hour) samples showed greatly increased levels of LC3B puncta staining compared to vehicle-treated sections, predominately in the tumor cell cytoplasm. (b) Time course of pS6 response following vehicle or MLN0128 treatment (5 mg/kg) of nude mice bearing HCT116 xenograft tumors. Images were analyzed by positive pixel analysis. The data indicated that pS6 was significantly reduced at all time points (*p* = 0.038). (c) Time course of LC3B response following vehicle or MLN0128 treatment (5 mg/kg) of nude mice bearing HCT116 xenograft tumors. Positive pixel image analysis showed increased LC3B expression within 30 minutes with Amp HQ staining (*p* = 0.029). (d) Western blot analysis indicating increasing levels of lipidated LC3B (LC3B-II) as early as 0.5 hours after exposure to MLN0128.

Calu-6 xenograft tumors were harvested from female nude mice following a single oral 5 mg/kg dose of MLN0128 and examined for levels of the mTOR pathway marker, pS6, using standard IHC and the autophagy marker LC3B using Amp HQ IHC. Within one hour of dosing, the level of pS6 was dramatically decreased (Figures 2(a) and 2(b)) indicating mTOR pathway inhibition. Quantification of pS6 with positive pixel analysis revealed a greater than 4-fold decrease (*p* = 0.038) in the MLN0128-treated tumor compared to the vehicle only control (Figure 2(b)). Also, a rapid increase in LC3B puncta in the cytoplasm of tumor cells from treated animals was observed(Figures 2(a) and 2(c)), indicating autophagy pathway induction. Quantification of LC3B puncta by positive pixel analysis revealed early and transient changes in signal levels at different time points (Figure 2(c)). The difference of the mean values of LC3B level between the animals treated with MLN0128 for 30 minutes and the vehicle group was statistically significant (*p* = 0.029). Consistent with the LC3B Amp HQ result, Western blot analysis of the HMF from the same tumor tissue also showed the lipidated LC3B increased after MLN0128 administration (Figure 2(d)).

### 3.3. Hypoxia-Induced Autophagy

**Figure 3:**
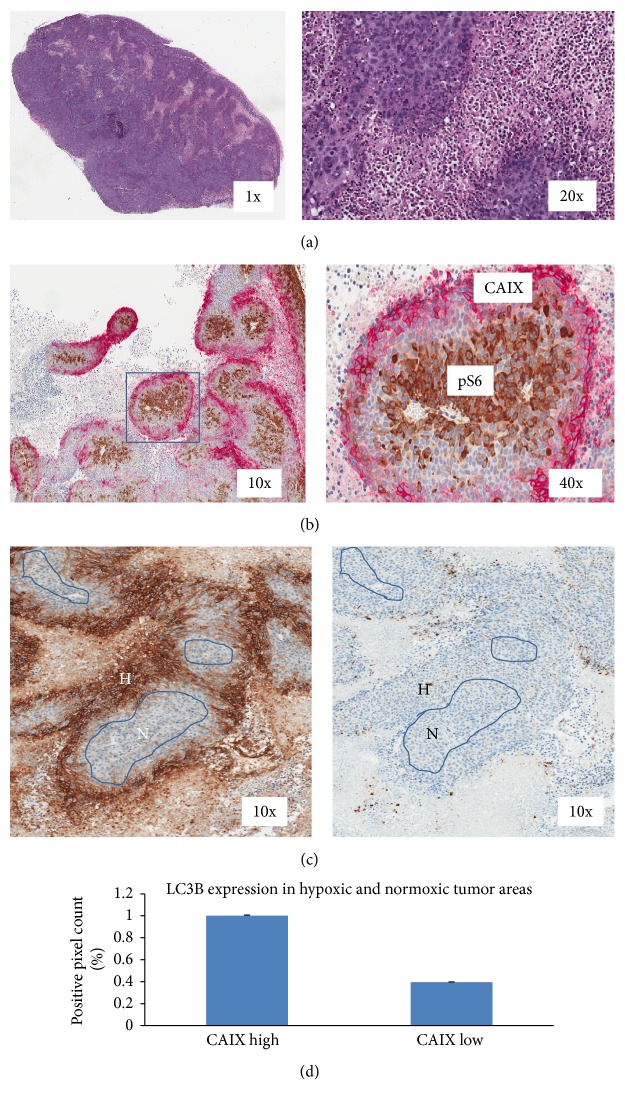
Comparison of LC3B levels in CAIX-positive (hypoxic) and CAIX-negative (normoxic) tumor areas. (a) H&E images Calu-6 xenograft tumor sections from vehicle-treated nude mice. (b) Standard IHC double labeling of CAIX (red) and pS6 (brown) in Calu-6 xenograft tumor sections from vehicle-treated nude mice. CAIX-positive staining (red stain) delineating hypoxic conditions was predominately observed in viable tumor adjacent to necrotic tumor areas (red stain), while the mTOR pathway marker pS6 showed positivity (brown stain) predominately in the normoxic region of tumor determined by CAIX-negative staining. There was very little overlap between the CAIX and pS6 marker staining. (c) Labeling of CAIX and LC3B (left image) was performed on separate Calu-6 xenograft serial sections to annotate CAIX-positive stained areas as hypoxic (H) and CAIX-negative areas as normoxic (N). CAIX-positive and CAIX-negative regions of the tumor sections were manually annotated on to LC3B stained slides indicated with blue lines. Inner circles within the viable tumor delineate areas with no CAIX staining were labeled as normoxic (N). Viable tumor positive for CAIX staining was labeled as hypoxic (H). (d) LC3B positive pixel image analysis results comparing hypoxic (H) regions with normoxic (N) tumor regions showed a significant difference in LC3B level between the two regions (*p* value = 0.012).

A central core of necrosis and areas of hypoxia are commonly observed in highly proliferating xenograft tumors. To investigate the relationship of hypoxia conditions with autophagy and mTOR pathway activity, Calu-6 tumors were analyzed for CAIX expression to annotate tumor hypoxic and normoxic regions and for levels of pS6 and LC3B to measure mTOR and LC3B pathway activities, respectively, using a dual IHC stain on a single slide. A high level of the hypoxia-induced protein CAIX expression was observed adjacent to the tumor necrotic areas indicating that necrosis in xenograft tumors was likely induced by hypoxia (Figure 3). Interestingly, with CAIX and pS6 double labeling, a dramatic decrease in pS6 signal in the strong CAIX staining hypoxic region was observed (Figure 3(b)), suggesting reduced mTOR signaling due to hypoxia. To assess autophagy activity in the context of hypoxia, sections of Calu-6 xenograft tumor were stained for CAIX and manually annotated as CAIX positive (hypoxic) and CAIX negative (normoxic). Annotated regions were subsequently transposed onto serial slides stained for LC3B (Figure 3(c)). The LC3B puncta in the CAIX-positive and CAIX-negative regions were measured using positive pixel image analysis. The areas of viable tumor with CAIX-positive staining had higher levels of LC3B puncta than CAIX-negative regions. The data showed a significant difference between the hypoxic region and the normoxic region (Figure 3(d)), when the two values were expressed as the mean ± SD (*p* = 0.012). One-way ANOVA was used for statistical analysis.

### 3.4. Downregulation of Autophagy Pathway following shRNA Knockdown of ATG7 in HCT116 Cells

**Figure 4:**
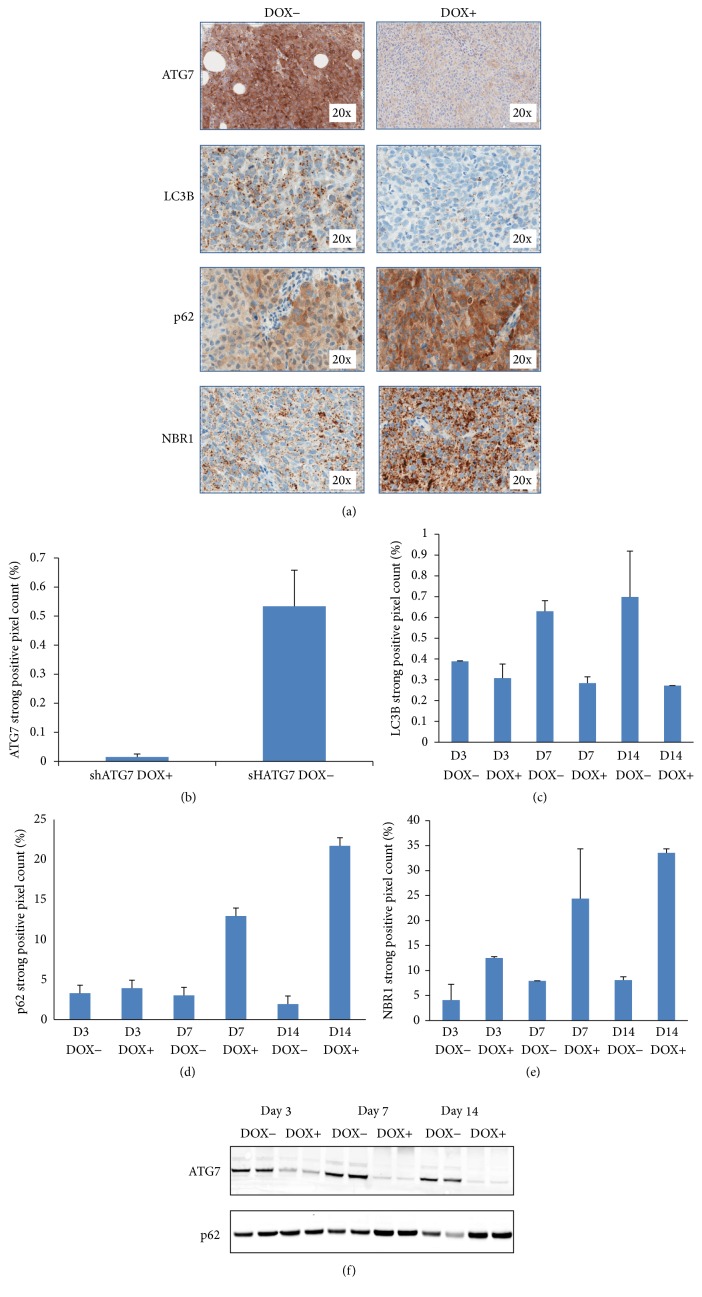
HCT116 doxycycline-inducible ATG7shRNA xenograft tumors were collected from tumor-bearing nude mice after 3, 7, and 14 days with or without doxycycline (DOX) treatment. One section from each tumor was stained for ATG7 and p62 by standard IHC methods and LC3B and NBR1 by Amp HQ IHC methods. Quantitative whole image analysis was performed using positive pixel count per total viable tumor area. (a) IHC images of ATG7, LC3B, p62, and NBR1 stained sections from xenograft tumors showed a decrease signal in ATG7 and LC3B accompanied by an increase of p62 and NBR1 signals after 14 days of DOX treatment compared with the control group (without DOX treatment). (b) Positive pixel analysis of sections stained for ATG7 by standard IHC showed a statistically significant decrease in ATG7 staining (*p* < 0.001) after 7 days of DOX treatment. (c) Positive pixel analysis of sections stained for LC3B with Amp HQ IHC showed a statistically significantly decrease in LC3B puncta staining after 7 and 14 days of DOX treatment (*p* < 0.030). (d) Positive pixel analysis of sections stained for p62 by standard IHC showed an increase in p62 expression after 7 and 14 days of DOX treatment. (e) Positive pixel analysis of sections stained for NBR1with Amp HQ showed a persistent increase after 3, 7, and 14 days of DOX treatment. (f) Consistent with IHC results, Western blot analysis showed a decreased intensity of ATG7 and an increased intensity of p62 with DOX-treated groups at days 3, 7, and 14 compared with the no DOX-treated groups.

ATG7 knockdown has been shown to impair formation of autophagosomes, the double-membrane vesicles which are responsible for delivering cytoplasm material to lysosomes [13]. Xenograft tumors were generated using HCT116 cells that express doxycycline-inducible ATG7-shRNA. A standard IHC with anti-ATG7 antibody staining was performed on the xenograft tumor collected at days 3, 7, and 14 after treatment with or without doxycycline. IHC images showed a dramatically reduced ATG7 protein expression in day 7 doxycycline-treated samples (Figure 4(a)). Positive pixel image analysis demonstrated a significant decreased expression of ATG7 in the doxycycline-treated tumors (*p* < 0.001) (Figure 4(b)). Further evaluation by Western blot analysis also showed decreased ATG7 protein levels with the doxycycline-treated group (Figure 4(f)). The same set of samples was analyzed using the Amp HQ method to detect LC3B puncta. As expected, doxycycline-induced ATG7 knockdown led to reduced LC3B expression (Figure 4(a)). Positive pixel algorithm analysis showed a greatly decreased LC3B expression in doxycycline-treated groups at 7 and 14 days (*p* = 0.030) (Figure 4(c)). As a result of ATG7 knockdown and impaired formation of autophagosomes, the accumulation of the adapter proteins p62 and NBR1 was detected in the doxycycline-treated samples (Figure 4(a)). Positive pixel algorithm analysis demonstrates a greatly increased p62 and NBR1 signal in doxycycline-treated groups on days 7 and 14 (Figures 4(d) and 4(e)). The Western blot analysis is consistent with the IHC results and also showed an increasing intensity of p62 on days 7 and 14 (Figure 4(f)).

### 3.5. Detection of LC3B and NBR1 in Xenograft Tumors

**Figure 5:**
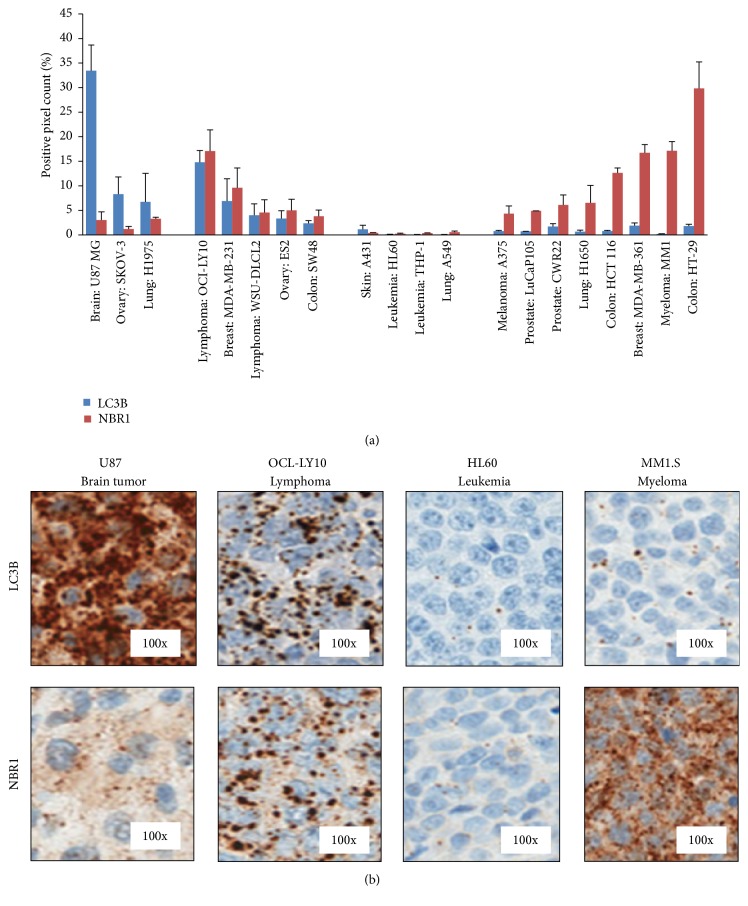
Xenograft tissues of multiple tumor types were randomly selected and stained in triplicate using LC3B and NBR1 Amp HQ IHC assays. (a) Whole slide image analysis was performed using positive pixel count. All values are expressed as the mean ± SD. The data show that the average basal expression of LC3B and NBR1 varied between tumors and the ratio of LC3B to NBR1 signal levels is not consistently proportional across the samples tested. U87 MG, SKOV-3, and H1975 xenograft tumors showed higher levels of LC3B compared to NBR1 in the same tumors. Conversely, A375, LuCap105, CWR22, H1650, HCT116, MDA-MD-361, MM1.S, and HT-29 xenograft tumors showed higher levels of NBR1 relative to LC3B. The remaining xenograft tumors were separated into groups having either relatively equivalent LC3B and NBR1 signals, including OCI-LY10, MDA-MB-231, WSU-DLCL2, ES2, and SW48, or very low expression of both markers including HL60, A549, and THP-1 xenograft tumors. (b) Representative images of LC3B and NBR1 stained xenograft tissues.

A panel of xenograft tumors was analyzed for LC3B and NBR1 using the Amp HQ method, as a way to assess the variability of basal autophagy activity across various tumor types. The data showed a wide range of basal LC3B and NBR1 signals across the various tumor models. These signal levels are not proportionally consistent across all samples tested. U87 MG (Brain) and SKOV-3 (Ovary) xenograft tumor models showed a high level signal of LC3B and low level of NBR1. HT-29 (Colon), MDA-MD-361 (Breast), MM1.S (Myeloma), and HCT116 (Colon) xenograft tumor models showed a high level signal of NBR1 and low level of LC3B. OCI-LY10 (Lymphoma) and MDA-MB-231 (Breast) xenograft tumor models showed a good correlation between the LC3B and the NBR1 signals. Xenograft tumor models, HL60 (Leukemia), A549 (Lung), and THP-1 (Leukemia), showed a very low signal for both markers (Figures 5(a) and 5(b)).

## 4. Discussion

Autophagy is an evolutionarily conserved process by which cytoplasmic proteins and organelles are catabolized. During starvation, the nutrient-responsive kinase mTOR is inhibited and a cell survival mechanism is upregulated that includes activating autophagy to reuse internal resources [7, 23, 24]. Using MLN0128 to inhibit mTOR activity and mimic starvation-induced autophagy, an increase was observed in LC3B puncta levels using tyramide-based signal amplification technology. The increased signal of the TSA method enabled the quantitative analysis of LC3B expression by an automated image analysis method. This was not possible with the traditional IHC method because of low specific signal and high background staining with the standard IHC. A downstream marker of the mTOR pathway, pS6, was used to monitor inhibition of mTOR following MLN0128 treatment. These data show a considerable decrease in pS6 levels starting as early as 0.5 hours after treatment. In response to mTOR inhibition, autophagy was activated and soluble LC3 (LC3B-I) was conjugated to form LC3-PE (LC3B-II) which participates in the formation of autophagosomes. Detecting LC3B by immunoblotting or immunofluorescence is commonly used methods for monitoring autophagy and autophagy-related processes [25].

In addition to direct mTOR inhibition, hypoxic conditions in the tumor microenvironment are also capable of rapidly inducing autophagy via induction of the hypoxia-inducible factor-1 (HIF-1) leading to mTOR inhibition [9, 10]. It is common to observe a central core of necrosis in a rapidly growing xenograft tumor where overexpression of carbonic anhydrase IX (CAIX) has been detected in hypoxic regions of tumors [26] and the CAIX IHC assay serves as a biomarker of hypoxia in many solid tumors. To understand the relationship between hypoxia and mTOR and autophagy pathway regulation, a colocalization analysis of CAIX with pS6 and LC3B was performed. In this study, CAIX-positive staining was observed in viable tumor adjacent to the tumor necrotic regions. A colocalization study with pS6 showed low pS6 levels in the same area that was positive for CAIX, demonstrating an inverse correlation between hypoxia and mTOR activity [27]. Further investigation using CAIX costaining with LC3B showed that there was higher level of LC3B in the CAIX-positive region, indicating hypoxia-induced autophagy in the hypoxic regions of the xenograft tumor.

A doxycycline-inducible ATG7shRNA model was used to study the dynamic range of the Amp HQ LC3B assay. Autophagy Ubl conjugation is a key feature of autophagosome formation and is mediated by the E1 enzyme ATG7. In this study, the intensity of ATG7 expression was drastically diminished in response to ATG7shRNA expression. As a result of ATG7 knockdown, a significant decrease in the number and size of the LC3B puncta was detected consistent with impaired autophagosome formation. This was accompanied by an increase in the level of the adaptor proteins NBR1 and p62. p62 is a multifunction protein [28] and, in the autophagy pathway, plays a role as a cargo receptor or adapter protein for degradation of ubiquitinated substrates. A direct interaction between the adapter proteins p62 and NBR1 and the autophagosomal protein LC3 results in aggregates which can be cleared through autophagy [14, 15]. Knocking down ATG7 caused impaired autophagy progression and stabilization of p62-positive bodies containing polyubiquitinated proteins. Using standard IHC and TSA methods, the accumulation of p62 and NBR1, respectively, was clearly detected. This data also showed a large dynamic range in LC3B and NBR1 levels in a panel of xenograft tumors, suggesting that the two markers were useful tools to support the intertumor assessment of autophagy pathway status. In conclusion, with this TSA technology, autophagy markers can be monitored in xenograft tumors which will be helpful in understanding the mechanism of autophagy regulation in tumor development.
